# Supplementary material for: In-sensor computing using a MoS2 photodetector with programmable spectral responsivity
Source: Nat Commun. 2023 Jul 17;14:4264. doi: 10.1038/s41467-023-40055-w (PMC10352275; doi:10.1038/s41467-023-40055-w)
Supplement: Supplementary file 1 — Supplementary Information [file 41467_2023_40055_MOESM1_ESM.pdf]

Supplementary Information for

In-sensor computing using a MoS_2 photodetector with programmable spectral responsivity

Dohyun Kwak‡, Dmitry K. Polyushkin‡ and Thomas Mueller*

Vienna University of Technology, Institute of Photonics, Gußhausstraße 27-29, 1040 Vienna, Austria

*Corresponding author: thomas.mueller@tuwien.ac.at

‡These authors contributed equally to this work.

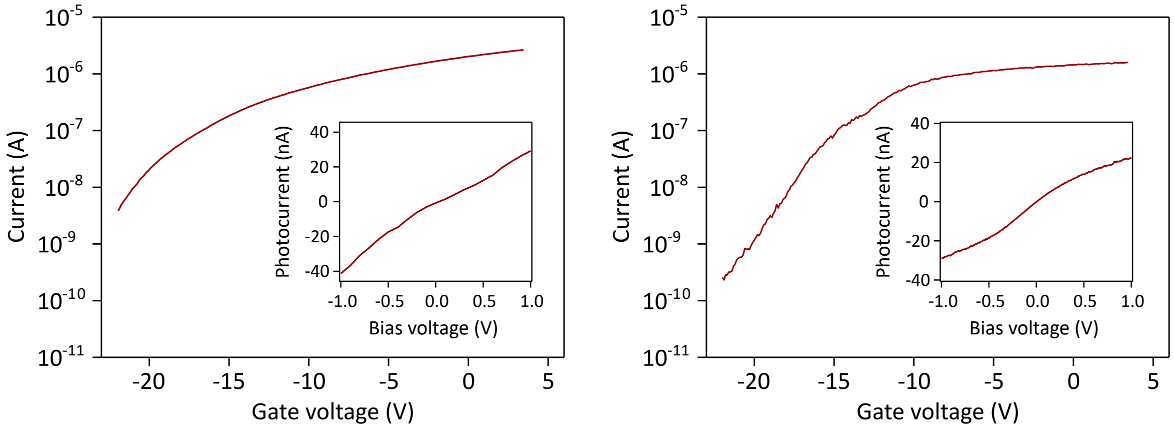

Supplementary Figure 1 | Electrical characteristics of other two detector segments.

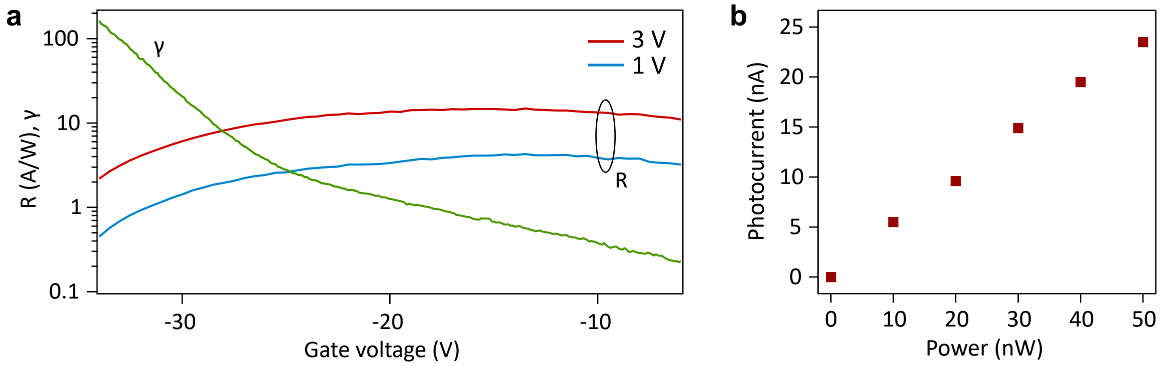

Supplementary Figure 2 | Gate and power-dependent photoresponse. a, The photoresponsivity R is shown for bias voltages of 1 V and 3 V in blue and red color, respectively. γ denotes the ratio between photocurrent and dark current at the light intensities typically used in the experiments. To sufficiently suppress the dark current, the measurements were performed at $V_G = -35$ V, albeit at the expense of a lower R. **b,** In the investigated light intensity range, the photoresponse exhibits a linear relationship with intensity.

```
import numpy as np
import matplotlib.pyplot as plt

# frequency-axis
f = np.linspace(300, 600, 200).reshape(-1,1)

# some generic spectra
u1 = 0.1 * np.sin(f/100)**2 + 0.3 + (f/10000)**2
u2 = 0.2 * np.sin(f/300)**2 + 0.3
u3 = 0.3 * np.sin(f/200)**2 + 0.2 # spectral signature of interest

# U-matrix
U = np.column_stack((u1,u2))

# some spectral mixture
a = 0.5
p = a*u3 + 0.5*(1-a)*u1 + 0.5*(1-a)*u2

# plot spectra
plt.plot(f, u3, label ='spectral signature of interest')
plt.plot(f, u1, label ='undesired spectral signature 1')
plt.plot(f, u2, label ='undesired spectral signature 2')
plt.plot(f, p, label ='measured spectrum')
plt.ylim(0, 0.55); plt.legend(); plt.show()

# calculate responsivity vector
r_T = u3.T.dot(np.identity(U.shape[0]) - U.dot(np.linalg.inv(U.T.dot(U)).dot(U.T)))

# plot responsivity vector
plt.plot(f, r_T.T, label ='photoresponsivity')
plt.legend(); plt.show()
```

Supplementary Note 1 | Python script for calculation of the responsivity vector in Figure 1.
